# Human milk-derived 5′-UMP promotes thermogenesis and mitochondrial biogenesis to ameliorate obesity

**DOI:** 10.3389/fnut.2025.1661778

**Published:** 2025-09-25

**Authors:** Ling Zhang, Zhou Peng, Caiyu Lin, Haixia Hu, Juan Du, Siqi Huang, Shan Huang, Jianfang Gao, Xirong Guo

**Affiliations:** ^1^Endocrinology Department, Tongren Hospital, Shanghai Jiao Tong University School of Medicine, Shanghai, China; ^2^Hongqiao International Institute of Medicine, Tongren Hospital, Shanghai Jiao Tong University School of Medicine, Shanghai, China

**Keywords:** 5′-UMP, human milk, maternal obesity, pyrimidine metabolism, childhoodobesity, thermogenesis, mitochondrial biogenesis

## Abstract

**Background:**

Traditional obesity interventions are often unsuitable for children. Breastfeeding has been shown to reduce obesity risk, potentially through bioactive metabolites in human milk. Here, we identified a human milk-derived metabolite, uridine 5′-monophosphate (5′-UMP), whose role in lipid metabolism and thermogenesis remains largely unknown.

**Methods:**

Untargeted metabolomics was performed on colostrum samples from obese and healthy mothers to identify obesity-associated metabolites. Zebrafish larvae and human preadipocytes were used to evaluate the anti-obesity effects of 5′-UMP. Lipid accumulation was assessed by Oil Red O and Nile Red staining, while mitochondrial function was analyzed using transgenic zebrafish [Tg(Xla. Eef1a1: mlsEGFP)] and fluorescent imaging.

**Results:**

Pyrimidine metabolism was significantly enriched in obese mothers, with orotate and 5′-UMP levels altered. Targeted analysis confirmed the presence of 5′-UMP in colostrum. Zebrafish toxicity assays confirmed 5′-UMP safety up to 200 μM. In the high-fat diet-induced zebrafish obesity model, 5′-UMP treatment significantly reduced abdominal lipid accumulation. In adipocytes, 5′-UMP enhanced mitochondrial respiration and increased mRNA and protein expression of PGC1-*α* and UCP1. Furthermore, mitochondrial fluorescence intensity and protein levels of NRF1 and MFN2 were elevated, indicating enhanced mitochondrial biogenesis and activity.

**Conclusion:**

Maternal obesity is associated with changes in the human milk metabolome. 5′-UMP, a nucleotide metabolite enriched in human milk, promotes thermogenesis and mitochondrial activation, effectively ameliorating obesity in zebrafish and human adipocytes. These findings support its potential as a safe, milk-derived therapeutic candidate for pediatric obesity intervention.

## 1 Introduction

Obesity, whose prevalence has increased over the last few decades to reach pandemic status, is a multifactorial pathology, influenced by environmental, genetic, and epigenetic factors (1, 2). This rapid increase in the prevalence of obesity may also result from early-life determinants (3). Among these determinants are maternal diet and neonatal feeding. Over half of pediatric cases progress to obesity, particularly affecting children in developing regions (4, 5), and predisposing them to early metabolic and cardiovascular comorbidities (6, 7). Maternal obesity is associated with changes in the human milk metabolome 8. While only a subset of metabolites correlated with maternal and infant weight, these point to potential milk-dependent mechanisms for mother–child transmission of obesity. Conventional weight reduction methods, including intermittent fasting, surgery, and dietary restrictions, are not suitable for use in children. Therefore, it is critical to identify safe and effective lipid-lowering components from human milk.

Recent studies show that breastfed children are less likely to develop obesity (8–10). In a cohort of 2,553 mother-infant pairs, breastfeeding is inversely associated with weight gain velocity and BMI (11, 12). Essential nutrients in human milk (13), such as peptides (14, 15), specific lipids (16), ribonucleotides (17), and nucleotides, protect infants from metabolic diseases. Pyrimidine metabolites have a complex relationship with lipid metabolism, which is regulated by an adipo-biliary-uridine axis (18). In addition to its role in nucleoside synthesis, uridine and its derivatives play an important role in glucose, lipid, and amino acid homeostasis (19). However, the role of pyrimidine uridine in lipid metabolism warrants further investigation. Promoting thermogenic adipocyte function to maintain lipid metabolism balance is critical for treating obesity and metabolic diseases (20, 21). Peroxisome proliferator-activated receptor gamma coactivator 1-alpha (PGC1-*α*) is a key coregulator in energy metabolism, maintaining body temperature and energy balance in the cold environment by upregulating the expression of uncoupling protein-1 (UCP-1) (22, 23). Stimulating PGC1-α and UCP-1 could be an effective strategy to prevent obesity.

The present study demonstrated that the pyrimidine metabolism pathway was associated with obese mothers using mass spectrometry. We identified uridine 5′-monophosphate (5′-UMP) as a key differential metabolite by untargeted and targeted metabolomic analyses of colostrum from obese and healthy mothers. We established a zebrafish obesity model and confirmed that 5′-UMP effectively reduced lipid accumulation. In parallel, experiments in human preadipocytes showed that 5′-UMP enhanced cellular energy metabolism and promoted mitochondrial biogenesis, collectively contributing to its anti-obesity effects.

## 2 Materials and methods

### 2.1 Clinical sample collection

Colostrum samples were collected at 3 days postpartum from lactating mothers who had given birth to term (37–41 weeks) infants at the Shanghai Tongren Hospital. Informed consent was obtained from all mothers. The study was approved by the Human Research Ethics Committee of Shanghai Tongren Hospital (Reference number 2021–086-02). Each sample was about 4 ~ 5 mL and collected on the third day postpartum. Samples were stored in sterile tubes at −80°C. We excluded mothers with diabetes, other medical conditions, or pregnancy complications. Participants were grouped by maternal prepregnancy. For the Chinese population, many studies reference the classification proposed by the China Working Group (24), which defines overweight as a BMI ≥ 24.0 kg/m^2^ and obesity as a BMI ≥ 28.0 kg/m^2^ (25, 26). Accordingly, we selected participants with BMI <24 kg/m^2^ (*n* = 6, lean) and ≥28 kg/m^2^ (*n* = 6, obese) for comparison.

### 2.2 Untargeted UPLC–MS/MS analysis

Calibra Lab at DIAN Diagnostics (Hangzhou, Zhejiang, China) conducted an untargeted metabolomic analysis on the CalOmics metabolomics platform. Samples were extracted using methanol (Sigma-Aldrich, United States) at a 1:4 ratio. The mixtures were shaken for 3 min and precipitated by centrifugation at 4,000 × g for 10 min at 20°C. Four aliquots of 100 μL supernatant were transferred to sample plates, dried under nitrogen, and re-dissolved in reconstitution solutions for UPLC–MS/MS analysis. The instrument for the UPLC–MS/MS methods are ACQUITY 2D UPLC (Waters, Milford, MA, United States) plus Q Exactive (QE) hybrid Quadrupole-Orbitrap mass spectrometer (Thermo Fisher Scientific, San Jose, United States). QE mass spectrometer was operated at a mass resolution of 35,000, the scan range was 70–1,000 m/z. Statistical analysis was performed using the R language (R version 3.4.1: http://www.cran.com; RStudio version 1.4.1717: https://www.rstudio.com; mixOmics version 6.10.9: http://mixomics.org/; randomForest version 4.6–14: https://www.rdocumentation.org/packages/randomForest/versions/4.6-14). Significantly changed metabolites between cases and control groups were identified using parametric (Student’s *t*-test, ANOVA) or non-parametric (Wilcox’s rank test, Kruskal-Wallis) methods.

### 2.3 Targeted metabolomics

Quantification of 5′-UMP (Yuanye, China; T94168; HPLC purity ≥99%) in human milk samples was performed using a CalQuant-S LC–MS/MS system. Data acquisition was conducted using Analyst software version 1.6.2, and quantitative analysis was performed using Multiquant software. An electrospray ionization source operated in positive ion mode was used. The scan mode was multiple reaction monitoring (MRM). Chromatographic separation was achieved on a C18 reverse-phase (UPLC BEH C18, 2.1×100 mm, 1.7 μm; Waters). The mobile phase consisted of (A) water containing 0.1% formic acid (Sigma-Aldrich, United States) and 2 mM ammonium acetate (Sigma-Aldrich, United States), and (B) acetonitrile (Sigma-Aldrich, United States) containing 0.1% formic acid. A gradient elution was employed over a total run time of 4 min.

Human milk samples collected from obese and normal-weight mothers were used for analysis. For each sample, 40 μL of milk was aliquoted, and an appropriate volume of protein precipitation solution containing internal standard was added. The mixture was vortexed thoroughly and centrifuged. The resulting supernatant was collected and subjected to LC–MS/MS analysis.

### 2.4 Zebrafish rearing and treatment

All animal experiments were approved by the Medical Ethics Committee of Shanghai Tongren Hospital of China (Approval No. A2022-017-01). Zebrafish (*Danio rerio*) were initially obtained from the China Zebrafish Resource Center and raised in the Hongqiao International Medical Research Institute, Shanghai Jiao Tong University School of Medicine. Wildtype zebrafish (Tuebingen, strain) and transgenic zebrafish [*Tg*(*Xla. Eef1a1:mlsEGFP*)] (27) were used in this study. All animal experiments were conducted following ARRIVE guidelines and regulations. Adult zebrafish were housed in an automated zebrafish system at 28.5°C in a 14-h light: 10-h dark cycle. Healthy fertilized zebrafish embryos at 24 h post-fertilization (hpf) were transferred into E3 embryo medium (5 mM NaCl, 0.17 mM KCl, 0.33 mM CaCl₂, 0.33 mM MgSO₄, and 0.1 μg/mL methylene blue) supplemented with 200 μM 1-phenyl-2-thiourea (PTU; Sigma, United States) to inhibit melanin pigmentation. Embryos were maintained in this medium until 7 days post-fertilization (dpf). In toxicity experiments, wildtype zebrafish embryos were randomly collected in E3 fish water with different concentrations (0 μM, 100 μM, 200 μM) of 5′-UMP from 8 hpf; each group contained approximately 50 embryos. Mortality, hatchability, and heart rate were assessed at 72 hpf and 96 hpf. As no toxicity was observed at the highest tested dose (200 μM) of 5′-UMP, this concentration was selected for subsequent experiments.

To establish a zebrafish obesity model (28), 0.5 g egg yolk powder (fat% ≥ 60; Company: Nanjing Ximenuo Biotechnology Co., LTD) was diluted in a 50 mL E3 medium. A high-fat diet (HFD) was prepared daily and fed for 6 h every day from 9 a.m. to 3 p.m. for 3 days, from 4 dpf to 7 dpf. 5′-UMP was diluted to the final concentration with E3 fish water containing PTU. From 4 to 7 dpf, zebrafish larvae were treated with 5′-UMP and fed for 6 h with HFD or a control diet. Zebrafish Lipid staining was analyzed at 18 h after the last day of feeding.

### 2.5 Zebrafish larvae lipid staining

On the 8 dpf, larvae were washed with PBS three times, fixed in 4% paraformaldehyde (PFA, Beyotime, P0099, China) overnight, and then washed with PBS three times. Larvae were fixed in 60% isopropanol for 30 min and stained with Oil Red O solution (0.3% Oil Red O in 60% isopropanol; Aladdin, China) for 2 h. After staining, larvae were washed alternately with PBS and observed after treatment under an Optec SZ760 stereomicroscope (Zeiss, Germany). Following the approach described in Karla Misselbeck et al. (29), the percentage of larvae exhibiting low, medium, and high lipid accumulation was calculated for each experimental group.

Nile Red staining was performed on 7 dpf zebrafish larvae (30). Zebrafish larvae were incubated with 0.5 μg/mL Nile Red solution (Aladdin, China) for 30 min in the dark, washed twice with E3 fish water, and imaged using the fluorescence microscope (Nikon, Japan).

### 2.6 Assessment of mitochondrial abundance in zebrafish following 5′-UMP treatment

To evaluate mitochondrial abundance *in vivo*, we used transgenic zebrafish larvae expressing mitochondrially targeted GFP [*Tg(Xla. Eef1a1:mlsEGFP)*]. Zebrafish were maintained under standard conditions and subjected to HFD feeding from 4 dpf to induce lipid accumulation. Larvae were randomly assigned to four groups: (1) control, (2) HFD, (3) HFD + 5′-UMP, and (4) 5′-UMP. At 7 dpf, larvae were anesthetized with tricaine (Sigma, USA, E10521) and imaged using a fluorescence microscope to capture GFP signals. Mitochondrial abundance was quantified based on mean fluorescence intensity using ImageJ software.

### 2.7 Cell culture

Human preadipocytes (ScienCell, United States, 7210/7220) were cultured in Preadipocyte Medium (PAM, ScienCell, United States) containing 5% fetal bovine serum (FBS), 1% penicillin–streptomycin (PS), and 1% preadipocyte growth supplement. Cells were maintained in a humidified incubator at 37°C with 5% CO₂. To induce differentiation, confluent preadipocytes were first cultured in DMEM/F12 medium (Gibco, United States) supplemented with 1 mM dexamethasone, 100 nM insulin, 1 μM rosiglitazone, 0.5 mM 3-isobutyl-1-methylxanthine (IBMX; all from Sigma, United States), and 1% PS for 4 days. Following induction, the cells were maintained in DMEM/F12 medium supplemented with 10 nM insulin and 1% PS for an additional 4 days, completing the 8-day differentiation protocol.

Cells were treated with 5′-UMP throughout the differentiation process. Control cells received an equal volume of 1% DMSO. Adipocyte differentiation efficiency was typically evaluated on Day 8 by performing Oil Red O staining (ScienCell, United States). The culture medium was refreshed every 2 days during the differentiation period.

### 2.8 Cell viability assay

The effect of 5′-UMP on the proliferation of human preadipocytes was evaluated by the CCK8 (Beyotime, China) assay. Briefly, cells were plated at 3.0 × 10^3^ density in a 100 μL volume in a 96-well plate. Then, adipocytes were treated for 0, 24, 48, and 72 h with various concentrations of 5′-UMP (0, 100, 200, and 400 μM). At each time, 10 μL of CCK-8 reagent was added to each well, followed by incubation at 37°C in a 5% CO₂ atmosphere for 1 h. Absorbance was measured at 450 nm using a microplate reader (Tecan, Swiss). Each condition was tested in 10 replicates.

### 2.9 Apoptosis analysis

Preadipocytes were treated for 48 h with various concentrations of 200 μM 5′-UMP. After that, the cells were harvested, washed, and resuspended in PBS. According to the manufacturer’s protocol, apoptotic cells were analyzed by staining cells with Annexin V-PE and 7-aminoactinomycin D (7-AAD; Vazyme, China). The cells were suspended in 1 × binding buffer at a 1.0 × 10^6^ cells/mL concentration. The cell suspension (100 μL) was then transferred to a 1.5-mL Eppendorf tube, mixed with 5 μL of Annexin V-PE and 10 μL of 7-AAD, and incubated for 20 min at RT in the dark. Within 1 h, the samples were analyzed using flow cytometry (BD, Canto II, United States). Data were analyzed using the FlowJo software.

### 2.10 Oil red O staining

Oil Red O staining kit (ScienCell, United States) was used to detect the lipid accumulation. Cells were fixed in 4% PFA for 15 min and stained with ORO solution (0.3% in isopropanol) for 30 min. After staining, the cells were washed 3 times and imaged using a microscope (Nikon, Japan).

### 2.11 Oxygen consumption rate

Oxygen consumption rate (OCR) was assessed using a Seahorse XF24 extracellular flux analyzer (Agilent, United States). Preadipocytes were plated in Seahorse XF 24-well microplates (Agilent, United States) and fully differentiated for 8 days. Subsequently, adipocytes were treated with 200 μM 5′-UMP for 24 h. Following treatment, adipocytes were incubated in a pre-warmed assay medium (XF base medium with 10 mM glucose, 1 mM sodium pyruvate, and 2 mM glutamine) for 1 h without CO_2_ before oxygen consumption analysis OCR was then assessed under basal conditions and after sequential injection of different reagents (103015–100, Agilent, United States), including 1.5-uM ATP synthase inhibitor oligomycin, 0.5 uM carbonyl cyanide 4-(trifluoromethoxy) phenylhydrazone, and 0.5-u rotenone/antimycin A. The OCR values were adjusted based on protein content.

### 2.12 RNA isolation and reverse transcription quantitative PCR

Total RNA was isolated from cultured cells using an RNA isolation reagent (Waltham, MA, United States). RNA purity and quantification were evaluated using the NanoDrop 2000 spectrophotometer (Thermo Scientific, United States). After RNA quantification, cDNA was synthesized using 1 μg of total RNA and a cDNA synthesis kit (Takara, Japan), according to the manufacturer’s instructions. Quantitative PCR (qPCR) was performed with a StepOnePlus Real-Time PCR system (ThermoFisher, Waltham, MA, United States) using a Premier qPCR kit (Thermo Scientific, Waltham, MA, United States). The expression levels were normalized to PPIA (Peptidyl-prolylcis-trans isomerase A) and analyzed by the 2^-ΔΔCt^ method. PPIA: F,5’-TTCATCTGCACTGCCAAGAC-3′; R,5’-TCGAGTTGTCCACAGTCAGC-3. UCP1: F,5’-AGGTCCAAGGTGAATGCCC-3′; R,5’-TTACCACAGCGGTGATTGTTC-3′. PGC1-*α*: F,5’-ACCTGACACAACACGGACAG-3′; R,5’-GTCTCCATCATCCCGCAGAT-3′.

### 2.13 Western blotting analysis

Cells were lysed in RIPA (Beyotime, China). Samples were separated by electrophoresis on 10% SDS-polyacrylamide gels and transferred to PVDF membranes. Membranes were blocked with 3% skimmed milk. After blocking, the membranes were incubated overnight at 4°C with antibodies against UCP1 (Proteintech, 23,673-1-AP, rabbit polyclonal, 1:3,000), Drp1 (Abcam, ab184247, rabbit monoclonal, 1:1,000), NRF1 (Proteintech, 12,482-1-AP, rabbit polyclonal, 1:1,500), MFN2 (Proteintech, 12,186-1-AP, rabbit polyclonal, 1:5,000), *β*-actin (Affinity Biosciences, AF7018, rabbit polyclonal, 1:1,000), followed by incubation with horseradish peroxidase-conjugated secondary antibodies (GeneTex, rabbit polyclonal, GTX213111-01, 1:1000) for 2 h.

### 2.14 Mito tracker staining

To assess mitochondrial abundance and activity, differentiated adipocytes were stained using MitoTracker™ Red CMXRos (ThermoFisher, Waltham, MA, United States). Before staining, cells were washed twice with PBS to remove the residual culture medium. The adipocytes were then incubated with MitoTracker working solution (final concentration: 100 nM) diluted in serum-free PAM for 40 min at 37°C in a humidified incubator containing 5% CO₂. After staining, cells were washed gently with PBS to remove excess dye and fixed with 4% paraformaldehyde for 15 min at room temperature. Fluorescent images were captured using a Nikon fluorescence microscope (Nikon, Tokyo, Japan), and mitochondrial fluorescence intensity was quantified using ImageJ software (NIH, United States). All experimental conditions were kept constant to ensure comparability between treatment groups.

### 2.15 Statistical analysis

Results are presented as the mean ± standard deviation (SD). Data were analyzed using *t*-tests in Prism 9 (version 9.0.0), and *p* < 0.05 was considered statistically significant.

## 3 Results

### 3.1 Untargeted metabolomic analysis of colostrum from obese and normal-weight mothers at 3 days postpartum

**Figure 1:**
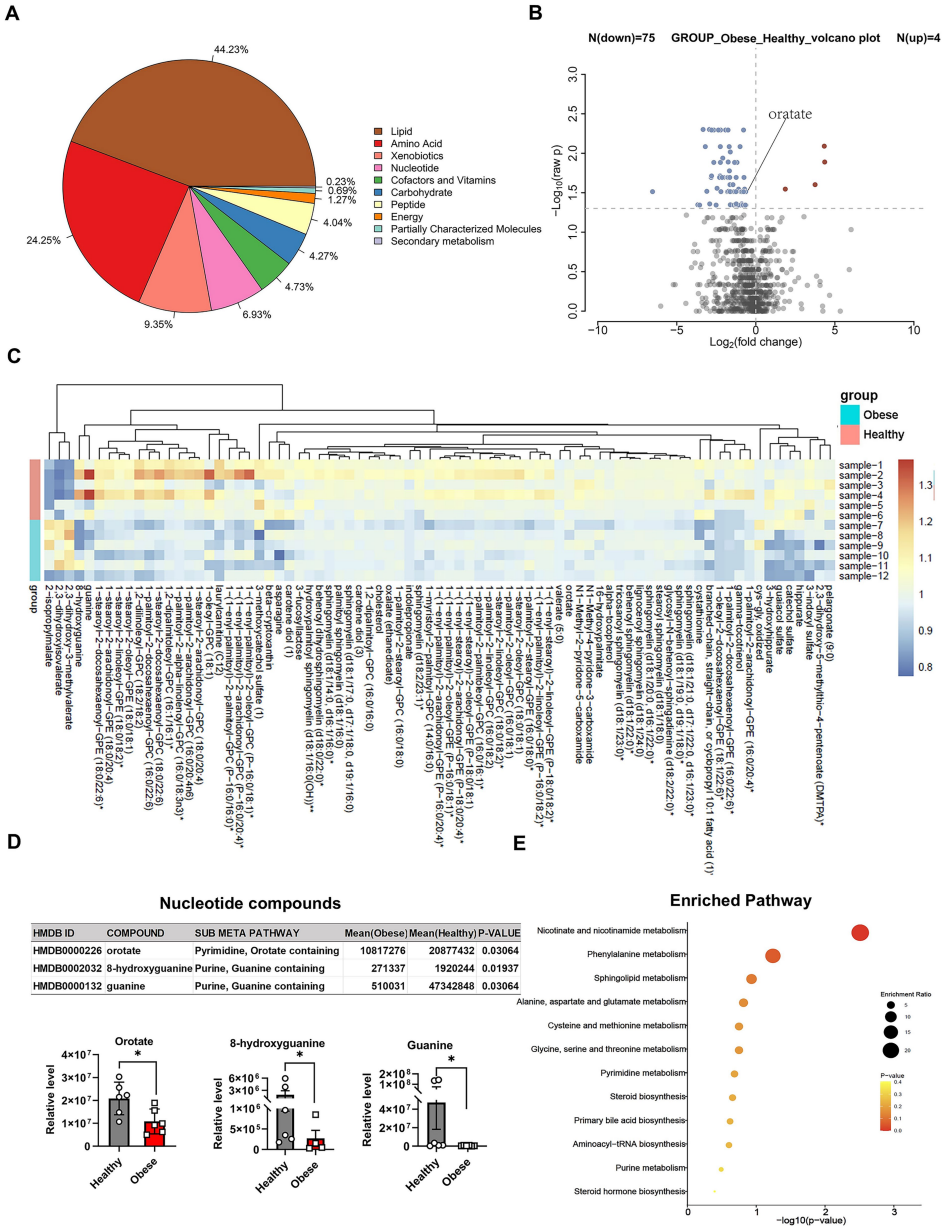
Metabolite differences in colostrum between the obese and control groups. **(A)** Proportional composition of colostrum metabolites categorized by super pathway. **(B)** Volcano plot showing differentially expressed colostrum metabolites between the obese and control groups (*p* < 0.05). **(C)** Heatmap of the top differentially expressed colostrum metabolites in the obese group compared to controls. **(D)** Relative levels of nucleotide-related metabolites (orotate, 8-hydroxyguanine, guanine). **(E)** KEGG pathway enrichment analysis of significantly altered metabolites.

Demographic and clinical information for the mother and infant is presented in Table 1. As expected, women in the obesity group had a significantly higher prepregnancy BMI than the normal group (28.79 ± 0.6387 kg/m^2^, compared with 21.83 ± 2.029 kg/m^2^, *p* < 0.0001). To investigate the impact of maternal body weight on colostrum composition, untargeted metabolomic profiling was performed on colostrum samples collected at 3 days postpartum from obese (*n* = 6, BMI ≥ 28 kg/m^2^) and normal-weight (*n* = 6, BMI < 24 kg/m^2^) mothers. The results showed that 866 metabolites were identified in both groups. As shown in Figure 1A, lipids accounted for the most significant proportion (44.23%), followed by amino acids (24.25%). Notably, nucleotide metabolites represented a considerable proportion (6.93%), indicating a significant enrichment of nucleotide-related compounds in colostrum (Figure 1A).

**Table 1:** Maternal and infant characteristics.

Characteristics	Normal (*n* = 6, BMI < 24)	Obesity (*n* = 6, BMI ≥ 28)	*p*-value
Infants’ characteristics at birth			
Gestational age (weeks)	39.33 ± 1.098	39.36 ± 0.8119	0.9668
Birth weight (g)	3,254 ± 325.9	3,590 ± 382.2	0.1325
Maternal characteristics			
BMI at birth (kg/m^2^)	21.83 ± 2.029	28.79 ± 0.6387	<0.0001
Milk collection time (postpartum days)	3	3	/

**Figure 2:**
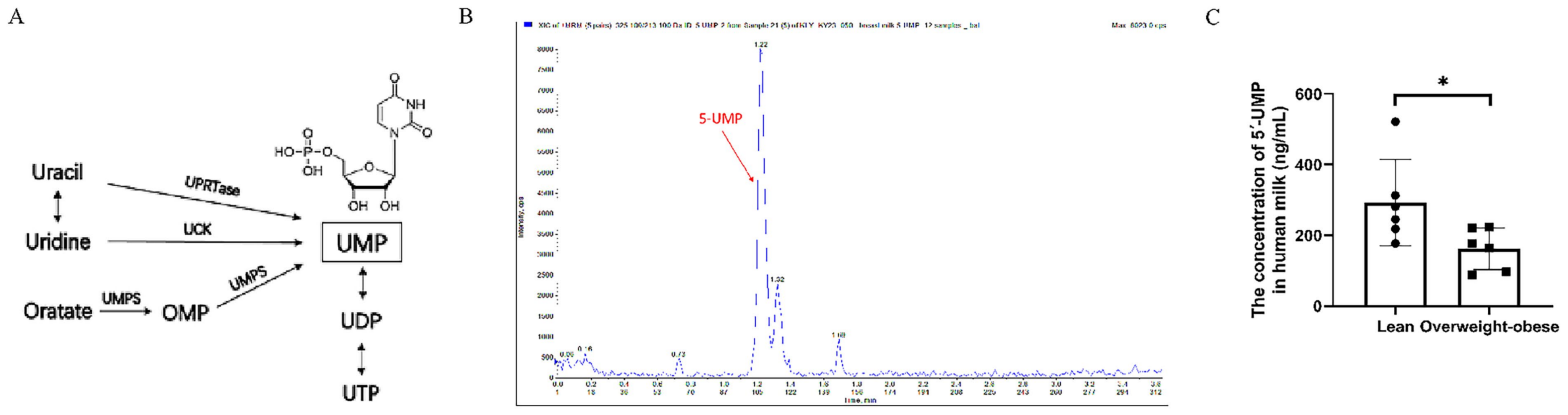
The levels of 5′-UMP in human colostrum. **(A)** Diagram of 5′-UMP synthesis pathways. **(B)** XIC diagram of 5’-UMP. **(C)** The concentration of 5′-UMP in colostrum from lean and overweight/obese mothers (ng/mL). 5′-UMP, uridine 5′-monophosphate.

Based on a significance threshold of *p* < 0.05, 79 metabolites were differentially expressed between the two groups (Figures 1B,C; Supplementary Table 1). Among them, three nucleotide-related metabolites were significantly downregulated in the colostrum of obese mothers compared to normal-weight controls: orotate (*p* = 0.0306), 8-hydroxyguanine (*p* = 0.0194), and guanine (*p* = 0.0306; Figure 1D). KEGG pathway enrichment analysis was then performed on the differentially expressed metabolites. Pyrimidine metabolism emerged as a significantly enriched pathway (Figure 1E). Notably, orotate (orotic acid), a key precursor in *de novo* pyrimidine biosynthesis, was enriched in this pathway and significantly reduced in the obese group (Figures 1B–E; Supplementary Table 2). These results suggest that orotate-related pyrimidine metabolism may be disrupted in obese mothers, play a crucial role in the pyrimidine pathway, and be associated with body weight regulation. Based on these findings, we further analyzed the metabolites of orotic acid and identified UMP as a key downstream metabolite (Figure 2A). Targeted metabolomic analysis was conducted on human milk samples from obese mothers and healthy controls (Figures 2B,C). The results revealed that the level of 5′-UMP was higher in the milk of normal-weight mothers compared to that of obese mothers. These findings suggest that 5′-UMP, as a downstream metabolite of orotic acid, may play a role in influencing offspring weight development.

### 3.2 Zebrafish-based toxicity test of 5′-UMP

**Figure 3:**
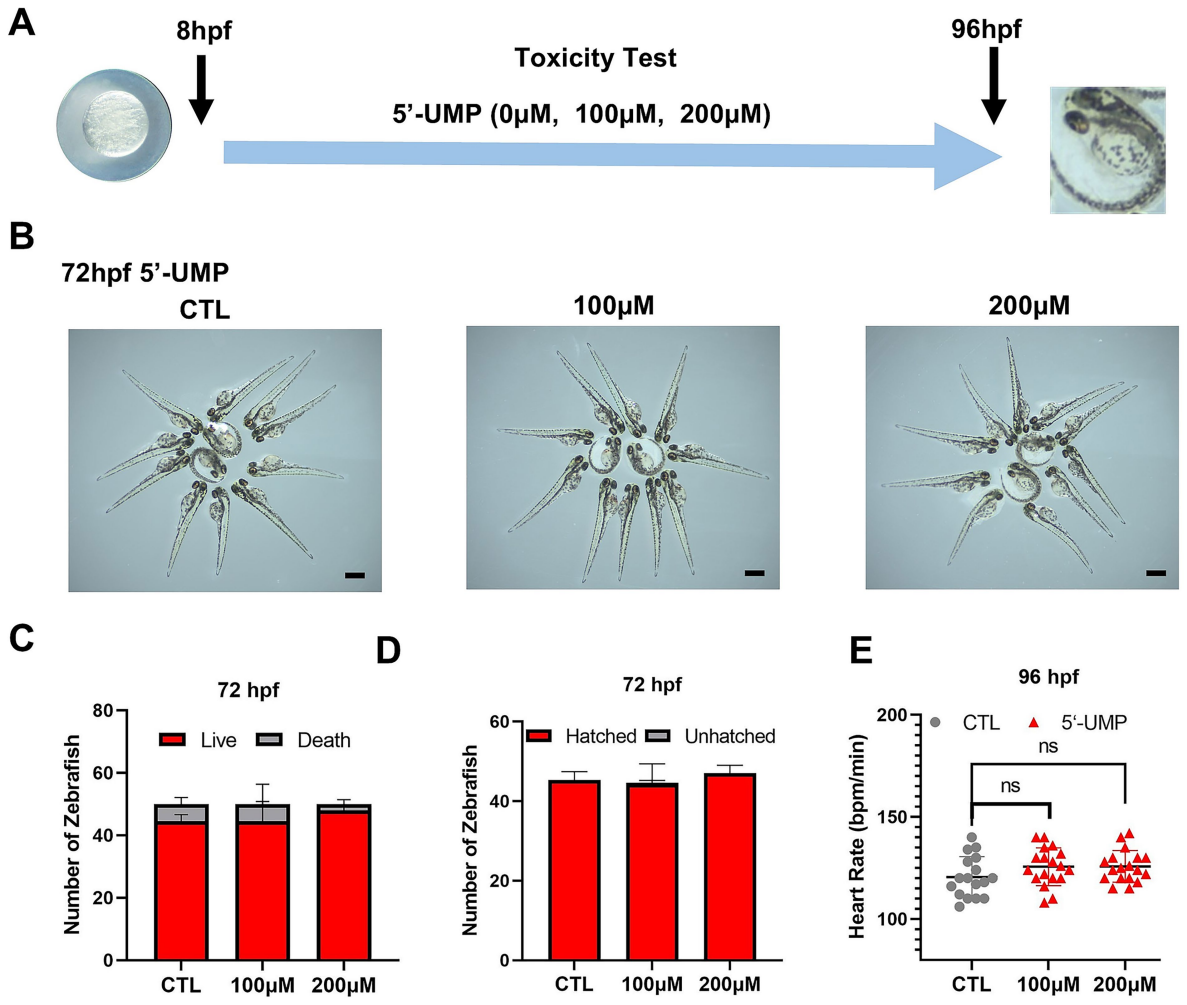
Toxicity test of 5′-UMP on the growth and development of zebrafish. **(A)** Schematic of the protocol for zebrafish in toxicity test and HFD-induced zebrafish model. 5’-UMP was added to embryos of zebrafish. **(B)** Morphology changes at 72 hpf, scale bar: 500 μm. **(C)** Mortality rate at 72 hpf. **(D)** Hatchability rate at 72 hpf. **(E)** Heart rate at 96 hpf. Data were obtained from three experiments (*n* > 30), and the graph shows the mean ± SD. 5′-UMP, uridine 5′-monophosphate; hpf, hours post-fertilization; dpf, days post fertilization; HFD, high-fat diet.

To investigate the potential effects of 5′-UMP on body weight, we conducted studies using a zebrafish model. As an initial step, a toxicity test on zebrafish embryos was undertaken to study the toxicity of 5′-UMP on zebrafish. Zebrafish larvae were exposed to increasing doses (0, 100, and 200 μM) of 5′-UMP during the first 3 dpf to determine the safe concentration. Given their high sensitivity to environmental stimuli, zebrafish serve as a reliable model for early toxicity assessment (Figure 3A). The results showed no significant differences in morphological changes (Figure 3B), survival rate (Figure 3C), hatching rate (Figure 3D), or heart rate (Figure 3E) at concentrations up to 200 μM 5′-UMP. Based on these results, 200 μM was determined to be a non-toxic concentration and was subsequently used for downstream functional analyses.

### 3.3 5’-UMP inhibits HFD-induced obesity in zebrafish

**Figure 4:**
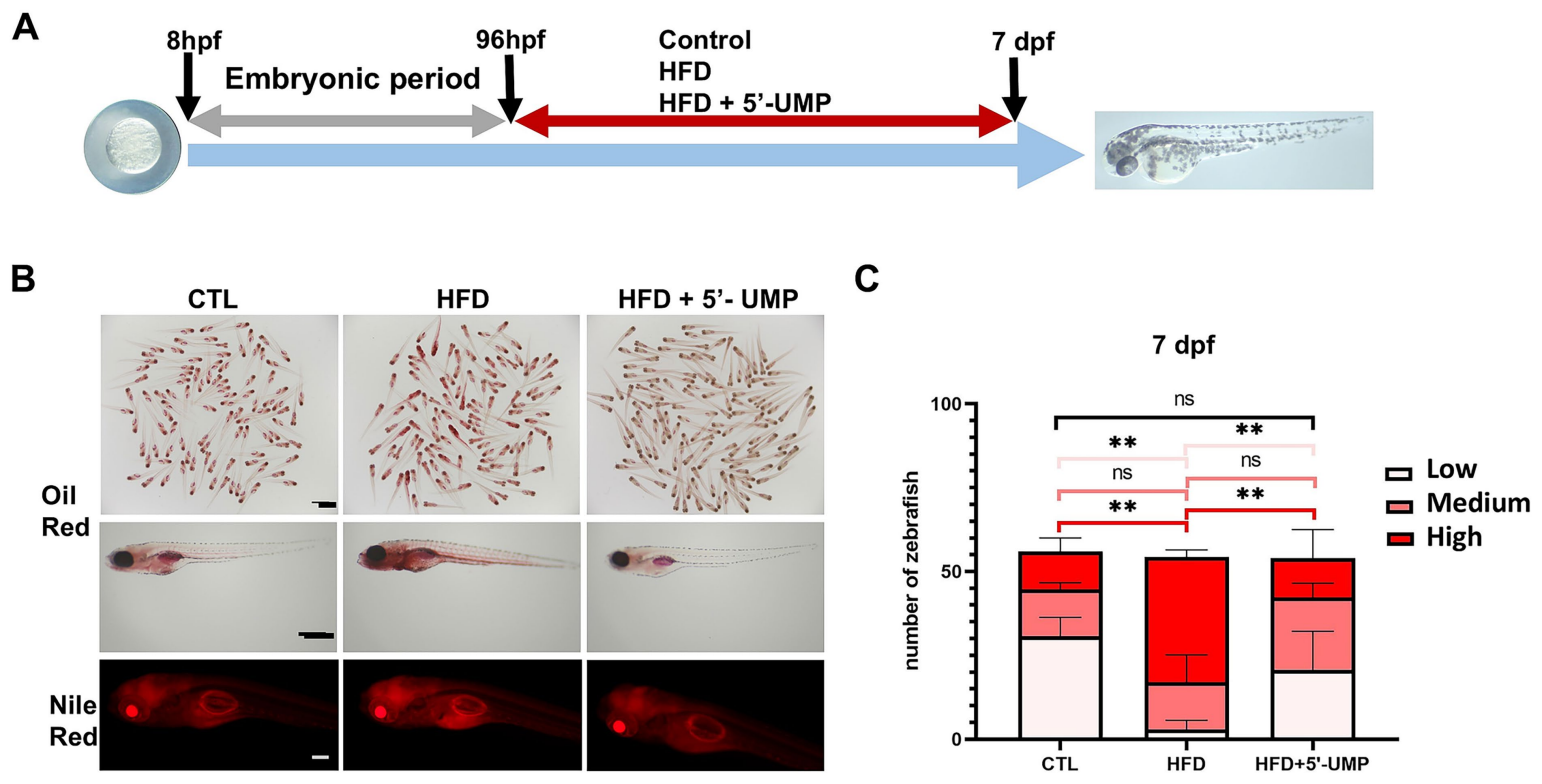
Effect of 5′-UMP on lipid accumulation in zebrafish larvae. **(A)** Schematic diagram of the experimental design. Schematic representation of the protocol used for examination of the effect of 5′-UMP treatment against HFD effects. Control: Zebrafish larvae were fed with a standard diet or HFD for 6 h each day from 4 to 7 dpf. 5′-UMP treatment: Zebrafish larvae were treated with 200 μM 5’-UMP during each control or HFD consumption period. **(B)** Oil Red O and Nile Red staining of zebrafish at 7 dpf under CTL, HFD, and HFD + 5′-UMP treatments. **(C)** Percentage of larvae with low, medium, and high lipid accumulation based on Oil Red O staining for the two indicated diets. All statistical analyses are presented on the right panel of data, which were pooled from three experiments (*n* = 51–58 per group); Data were obtained from three experiments, and the graph shows the mean ± SD. **p < 0.01; ns, not significant. 5′-UMP, uridine 5′-monophosphate; dpf, days post fertilization; HFD, high-fat diet.

The HFD-induced zebrafish model was used to validate the potential effect of 5′-UMP. Zebrafish larvae were divided into control, HFD, and 5′-UMP-treated HFD zebrafish. After 4 days of rearing, the zebrafish larvae were fixed and stained (Figure 4A). As shown in Figure 4B, Oil Red O and Nile Red staining revealed a marked increase in lipid deposition in HFD-fed larvae compared to the control group. In contrast, 5′-UMP treatment visibly reduced lipid accumulation, as evidenced by weaker Oil Red O staining and decreased Nile Red fluorescence intensity. Quantitative analysis on Day 7 further confirmed this observation (Figure 4C): HFD feeding significantly increased the number of zebrafish with high lipid content (*p* < 0.01), whereas 5′-UMP administration led to a shift toward lower lipid categories, with a significantly reduced proportion of high-lipid individuals (*p* < 0.01) and an increased proportion in the low-lipid group. These results indicate that 5′-UMP effectively attenuates lipid accumulation *in vivo* under HFD exposure.

### 3.4 5′-UMP attenuates lipid accumulation in adipocytes

Inhibiting adipogenesis and promoting the thermogenesis of adipocytes are two strategies against obesity. To evaluate the adipocyte cytotoxicity of 5′-UMP, we tested the viability using the CCK8 for preadipocytes exposed to graded concentrations of 5′-UMP (0, 100, 200, 400 μM) for 24, 48, and 72 h calculated as a percentage of control, untreated cells. The concentrations of 400 μM 5′-UMP and lower did not cause any changes in the relative cell viability (Supplementary Figure S1A). Then, we evaluated the apoptosis of human preadipocytes pretreated with 200 μM 5′-UMP for the indicated times. The flow cytometry-based apoptosis analysis exhibited no differences after 48 h treatment compared to that in the non-treated cells (Supplementary Figure S1B). These findings suggest that the selected dose of 5′-UMP was safe for preadipocytes.

**Figure 5:**
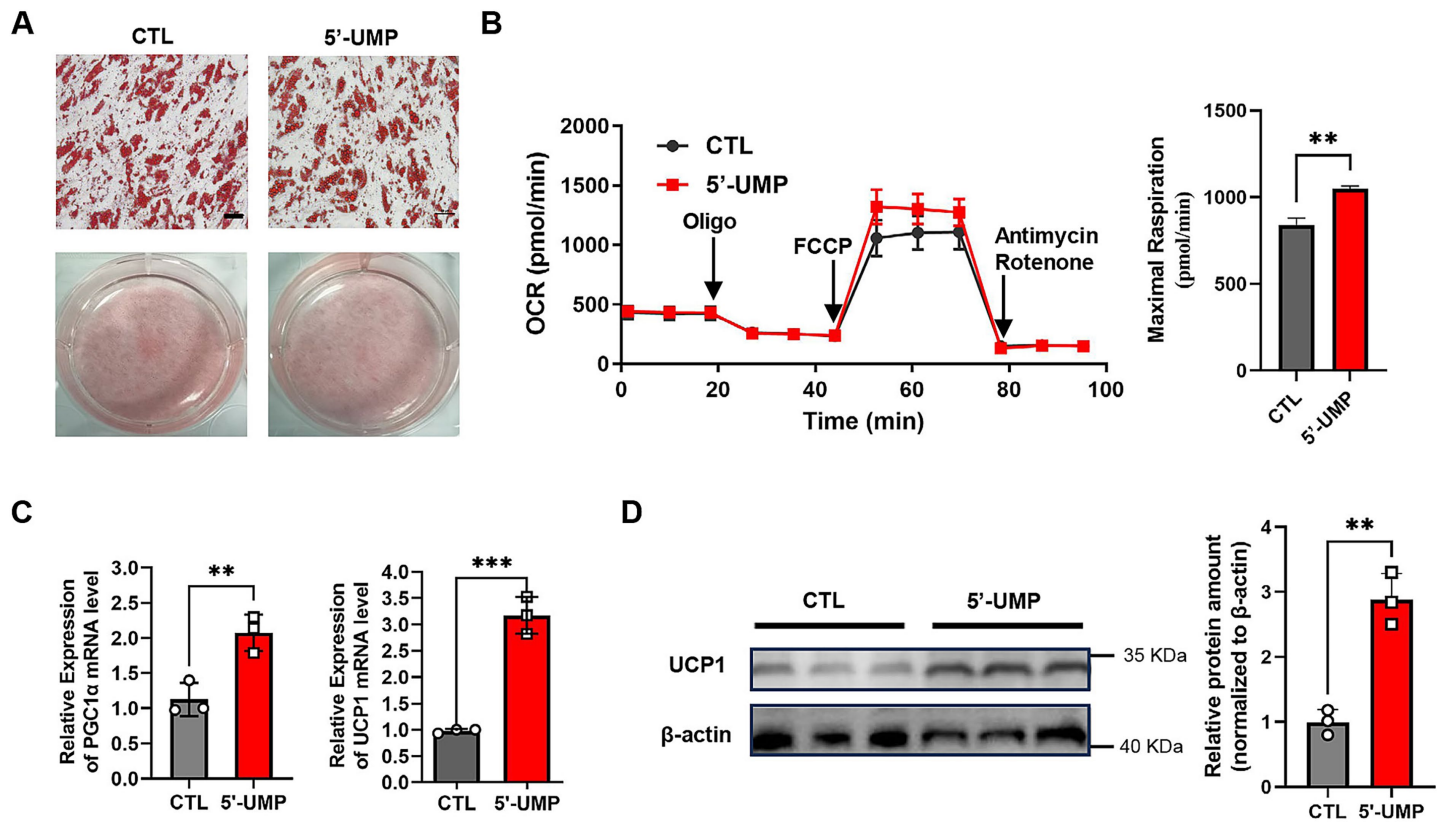
The effect of 5′-UMP on thermogenesis in adipocytes. **(A)** Lipid accumulation was detected by Oil Red O staining, and a representative image of the staining was shown by optical microscopy at ×200 magnification, scale bar: 50 μm. **(B)** Oxygen consumption rate (OCR) measurement and bar graph of maximal respiration. Quantitative analysis of maximal respiration of 5’-UMP compared to the control. **(C)** Quantified PCR was collected with 200 μM 5’-UMP compared to the control. **(D)** Immunoblot and quantification of UCP1 protein levels normalized to *β*-actin. Data are presented as mean ± SD. The protein was collected in three independent experiments. *p < 0.05, **p < 0.01, ***p < 0.001.

To explore the cellular effects of 5′-UMP on lipid metabolism and mitochondrial function, human preadipocytes were treated with 5′-UMP during adipogenic differentiation. As shown in Figure 5A, Oil Red O staining revealed reduced intracellular lipid accumulation in 5′-UMP-treated cells compared to the control group, suggesting an inhibitory effect on adipogenesis. Mitochondrial respiration was assessed using Seahorse extracellular flux analysis. As shown in Figure 5B, 5′-UMP treatment significantly enhanced maximal OCR following FCCP stimulation (*p* < 0.01), indicating improved mitochondrial oxidative capacity. In line with this observation, thermogenic and mitochondrial biogenesis marker expression levels were significantly upregulated upon 5′-UMP treatment. Specifically, mRNA expression of PGC1α and UCP1 was markedly elevated (*p* < 0.01, *p* < 0.001, respectively; Figure 5C). Western blot analysis further confirmed increased UCP1 protein levels in the 5′-UMP group (Figure 5D), supporting the induction of a thermogenic gene program.

Collectively, these results demonstrate that 5′-UMP not only reduces lipid accumulation but also promotes thermogenic gene expression in human adipocytes.

### 3.5 5′-UMP activates mitochondrial biogenesis and dynamics *in vitro* and in vivo

**Figure 6:**
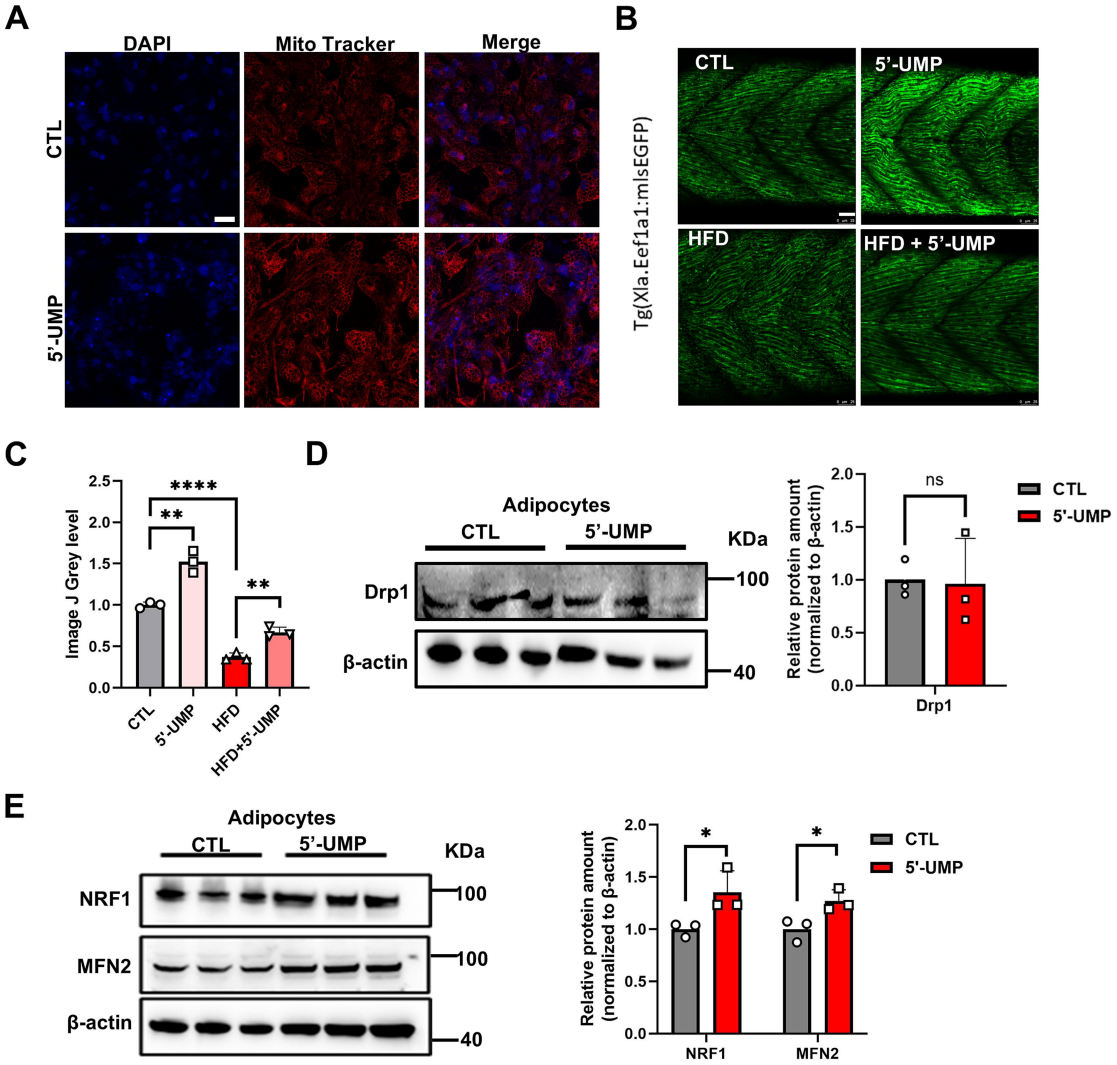
The influence of 5’-UMP on mitochondrial biogenesis. **(A)** Representative images of Mito tracker in human primary visceral preadipocytes, scale bar: 50 μm. **(B)** Zebrafish *(Tg(Xla. Eef1a1:mlsEGFP))* were fed a high-fat diet, and a representative image was shown by fluorescence microscope, scale bar: 25 μm. **(C)** Quantitative analysis of fluorescence intensity was shown. **(D)** Immunoblot of DRP1 in treated mature human adipocytes. The quantitative analysis was shown as a bar graph in the right panel (*n* = 3). **(E)** Immunoblot of NRF1 and MFN2 in treated mature human adipocytes. The quantitative analysis was shown as a bar graph in the right panel (*n* = 3). Data are presented as mean ± SD. The protein was collected in three independent experiments. *p < 0.05, **p < 0.01, ****p < 0.0001.

To evaluate the effects of 5′-UMP on mitochondrial function, we performed MitoTracker staining in adipocytes. As shown in Figure 6A 5′-UMP treatment led to a marked increase in mitochondrial fluorescence intensity compared to the control group, suggesting enhanced mitochondrial content or activity.

Next, we utilized transgenic zebrafish larvae expressing mitochondrially targeted GFP [*Tg(Xla. Eef1a1:mlsEGFP)*] to evaluate the effect of 5′-UMP on mitochondrial abundance under HFD conditions. As shown in Figures 6B,C, mitochondrial fluorescence was significantly reduced in the HFD group, while 5′-UMP administration restored the fluorescence intensity to near-control levels (*p* < 0.01). Notably, zebrafish treated with 5′-UMP alone exhibited even higher mitochondrial fluorescence than controls (*p* < 0.01), indicating a mitochondrial-boosting effect of 5′-UMP in both physiological and HFD-induced conditions. We further analyzed the expression of mitochondrial biogenesis and fusion-related proteins to investigate the underlying mechanisms. Western blot analysis showed that 5′-UMP did not significantly alter the mitochondrial fission protein Drp1 expression in adipocytes (Figure 6D). In contrast, the expression of mitochondrial biogenesis regulator NRF1 and the mitochondrial fusion protein MFN2 was significantly upregulated following 5′-UMP treatment (Figure 6E), indicating enhanced mitochondrial biogenesis and fusion. Together, these results suggest that 5′-UMP enhances mitochondrial activity by promoting mitochondrial biogenesis and fusion, potentially through upregulation of NRF1 and MFN2.

## 4 Discussion

Early-life determinants are considered a significant factor in the rapid increase of obesity. However, while maternal nutrition has been extensively studied, the effects of breastfeeding on the reprogramming of energy balance in childhood and throughout adulthood remain largely unknown. Previous studies combined detailed metabolomic analysis of human milk from lean compared with overweight or obese women, together with longitudinal analysis of weight gain and body composition in infants, demonstrated that differentially abundant metabolites in milk are associated with infant obesity (31, 32). These data indicate that obesity-associated differences in human milk composition might contribute to early childhood obesity. In this study, we demonstrated that 5’-UMP, a pyrimidine nucleotide metabolite enriched in human milk, may serve as a protective factor against early-life obesity by promoting thermogenesis and mitochondrial biogenesis. Through metabolomic profiling of colostrum from obese and normal-weight mothers, we identified alterations in the pyrimidine metabolism pathway, specifically decreased levels of 5’-UMP in the milk of obese mothers. The developmental stage of zebrafish larvae is analogous to infancy and early childhood in humans. Therefore, Functional experiments in zebrafish larvae and human preadipocytes further demonstrated the lipid-lowering and thermogenic properties of 5’-UMP, highlighting that 5’-UMP may be a potential therapeutic metabolic from pyrimidine nucleotides for treating obesity.

Previous studies investigating the human milk metabolome primarily focused on gestational age, lactation stage, or maternal health status, such as gestational diabetes mellitus and dietary patterns (33, 34). Although the overlap between metabolites associated with maternal BMI and infant adiposity was limited (31), studies have reported that breast milk or its derived components may benefit weight loss or metabolic health. For example, maternal exercise increases the level of 3′-sialyllactose in breast milk, directly improving offspring metabolism by enhancing glucose regulation and reducing fat accumulation (32). Additionally, breast milk alkylglycerols—unique lipid signaling molecules essential for healthy adipose tissue development—promote the maintenance of beige adipocytes via interactions with adipose tissue macrophages (16). Another human milk key component, the oligosaccharide 2′-fucosyllactose (2′-FL), has been shown to protect against high-fat diet-induced obesity in mice. This effect is attributed to its ability to modulate intestinal mucus production, composition, and degradation, which are linked to alterations in gut microbiota and fecal proteome profiles (35).

Although the overlap between metabolites associated with maternal BMI and infant adiposity was limited (31). More recently, several nucleoside and nucleotide derivatives, such as orotate, adenosine 3′,5′-cyclic monophosphate, and cytidine, were differentially abundant in milk from obese women compared with normal weight women at 1 month (31), raising the question of whether human milk nucleotide content may affect systemic metabolism in the infant. Our study demonstrates that maternal obesity is modestly associated with changes in the milk metabolome, notably characterized by a significant decrease in orotate levels. Notably, the pyrimidine nucleotide uridine has been discovered to regulate thermogenesis during the fasting–feeding transition (18). However, the functional significance of these nucleotide components remains poorly understood.

In our research, we revealed that the level of 5′-UMP in the breast milk of normal-weight mothers was higher than that in obese mothers. As a central metabolite in pyrimidine metabolism, 5′-UMP is produced in the cytosol from orotate through a two-step reaction catalyzed by UMP synthetase. It is essential for *de novo* and salvage synthesis pathways and gives rise to all other pyrimidine nucleotides (36). Our findings that 5′-UMP enhances mitochondrial function and thermogenic gene expression in adipocytes align with previous evidence showing disrupted pyrimidine synthesis and reduced 5′-UMP levels in obesity. Exogenous 5′-UMP has been reported to counteract obesity by restoring ceramide balance via the HIF2*α*–ACER2 pathway (37).

Inhibiting adipocyte adipogenesis and enhancing the thermogenic capacity of adipocytes are primary strategies for treating obesity. In a HFD zebrafish model, 5′-UMP reduced lipid accumulation in the abdomen. Mechanistically, we observed that 5’-UMP increased mitochondrial fluorescence intensity in both zebrafish larvae and human adipocytes, accompanied by upregulation of NRF1 and MFN2, key regulators of mitochondrial biogenesis and dynamics. These findings imply that 5’-UMP exerts its anti-obesity effects, at least partially, by enhancing mitochondrial capacity and promoting oxidative metabolism. Given that mitochondrial dysfunction is a hallmark of obesity and metabolic diseases, the ability of 5’-UMP to restore mitochondrial activity presents a promising avenue for intervention. Similar species-specific effects have been observed with orotate, a precursor in pyridine biosynthesis. When orotate is administered to mice, it can induce hepatic steatosis with a certain probability, but this effect is not observed in rats (38, 39). These results suggest that 5′-UMP promotes thermogenesis by enhancing mitochondrial biogenesis and maintaining mitochondrial homeostasis.

Although our study primarily focuses on the early-life implications of 5′-UMP as a breast milk–derived metabolite, accumulating evidence suggests its potential benefits in adult obesity. A recent study demonstrated that UMP levels are markedly reduced in obese adults and obese mice, and that dietary supplementation with UMP alleviates obesity-related phenotypes in adult mice, including reductions in body weight, liver mass, and adiposity, along with improvements in energy metabolism and insulin sensitivity (40). Thus, 5′-UMP, as a potential therapeutic agent, also highlights its broader therapeutic potential in adult obesity. Our study has several implications. First, it highlights a milk-derived nucleotide as a safe and bioactive factor potentially contributing to obesity prevention, especially during the critical window of infancy when energy metabolism is highly plastic. However, some limitations warrant mention. The sample size for human milk metabolomics was modest, and larger cohorts are needed to validate our findings across diverse populations. Additionally, while our *in vivo* and *in vitro* models provide mechanistic insights, longitudinal studies in human infants will be necessary to confirm the causal relationship between 5’-UMP intake and obesity prevention. Furthermore, we did not assess other milk components, such as immune cells, microbiota, or hormones. That may also contribute to offspring metabolic outcomes.

## 5 Conclusion

Maternal obesity is associated with metabolomic signatures in human milk. Our findings indicate that 5′-UMP, derived from the pyrimidine metabolism pathway, is associated with obese mothers, as identified through mass spectrometry. 5′-UMP reduces abdominal lipids in zebrafish larvae fed a HFD and influences adipocyte thermogenesis. The underlying mechanisms suggest that 5’-UMP may promote mitochondrial biogenesis via the PGC1-α/NRF1 pathway. Therefore, 5’-UMP is considered a safe and therapeutic metabolite from breast milk for preventing and treating obesity.

## Data Availability

The original contributions presented in the study are publicly available. This data can be found here, Accession Number: MTBLS13027, https://www.ebi.ac.uk/metabolights/editor/MTBLS13027/descriptors.

## Supplementary material

SUPPLEMENTARY FIGURE S1 The proliferation and apoptosis of preadipocytes upon 5′-UMP treatment. **(A)** Effect of 5′-UMP on proliferation at the concentration of 100 μM, 200 μM, and 400 μM. **(B)** Effect of 5′-UMP on apoptosis at a concentration of 200 μM.
