# Supplementary figures and images for: Human milk-derived 5′-UMP promotes thermogenesis and mitochondrial biogenesis to ameliorate obesity
Source: Front Nutr. 2025 Sep 25;12:1661778. doi: 10.3389/fnut.2025.1661778 (PMC12509697; doi:10.3389/fnut.2025.1661778)

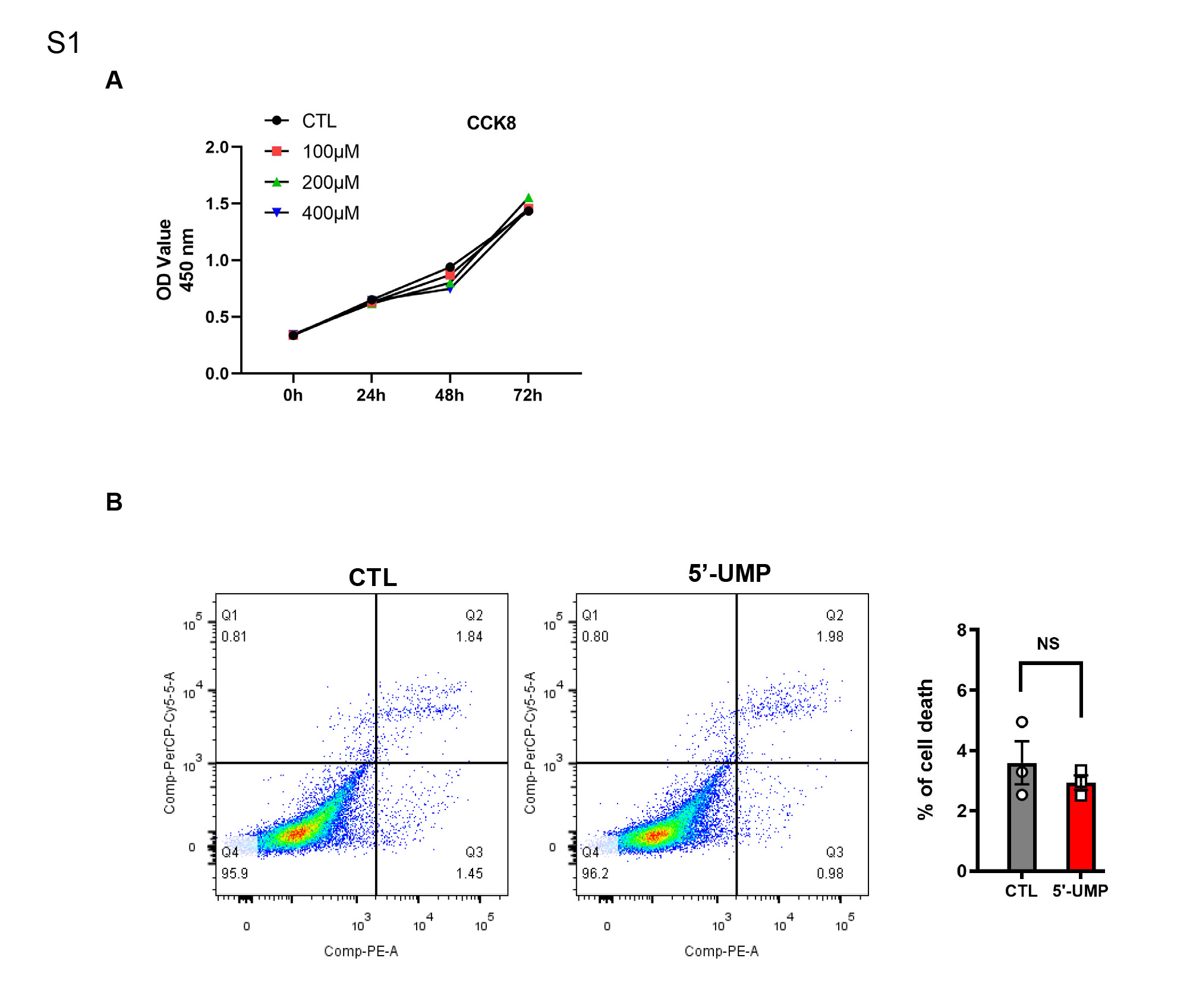

Supplement: SUPPLEMENTARY FIGURE S1 — The proliferation and apoptosis of preadipocytes upon 5′-UMP treatment. (A) Effect of 5′-UMP on proliferation at the concentration of 100 μM, 200 μM, and 400 μM. (B) Effect of 5′-UMP on apoptosis at a concentration of 200 μM. [file Image_1.jpeg]
