# Supplementary material for: Human milk-derived 5′-UMP promotes thermogenesis and mitochondrial biogenesis to ameliorate obesity
Source: Front Nutr. 2025 Sep 25;12:1661778. doi: 10.3389/fnut.2025.1661778 (PMC12509697; doi:10.3389/fnut.2025.1661778)
Supplement: Supplementary file 3 [file Table_2.docx]

| **Pathway** | **Pathway ID** | **total** | **expected** | **hits** | **Raw.p** | **Holm.p** | **FDR** | **Enrichment Ratio** | | **log.P.value** | **metabolite.hits** | |
| --- | --- | --- | --- | --- | --- | --- | --- | --- | --- | --- | --- | --- |
| Nicotinate and nicotinamide metabolism | hsa00760 | 15 | 0.0879 | 2 | 0.00308 | 0.259 | 0.259 | 22.7531 | 2.51144928 | | | N1-Methyl-4-pyridone-3-carboxamide, N1-Methyl-2-pyridone-5-carboxamide |
| Phenylalanine metabolism | hsa00360 | 10 | 0.0586 | 1 | 0.0572 | 1 | 1 | 17.0648 | 1.24260397 | | | Hippuric acid |
| Sphingolipid metabolism | hsa00600 | 21 | 0.123 | 1 | 0.117 | 1 | 1 | 8.13008 | 0.93181414 | | | SM(d18:1/18:0) |
| Alanine, aspartate and glutamate metabolism | hsa00250 | 28 | 0.164 | 1 | 0.153 | 1 | 1 | 6.09756 | 0.81530857 | | | L-Asparagine |
| Cysteine and methionine metabolism | hsa00270 | 33 | 0.193 | 1 | 0.178 | 1 | 1 | 5.18135 | 0.74958 | | | L-Cystathionine |
| Glycine, serine and threonine metabolism | hsa00260 | 33 | 0.193 | 1 | 0.178 | 1 | 1 | 5.18135 | 0.74958 | | | L-Cystathionine |
| Pyrimidine metabolism | hsa00240 | 39 | 0.229 | 1 | 0.207 | 1 | 1 | 4.36681 | 0.68402965 | | | Orotic acid |
| Steroid biosynthesis | hsa00100 | 42 | 0.246 | 1 | 0.221 | 1 | 1 | 4.06504 | 0.65560773 | | | Cholesterol |
| Primary bile acid biosynthesis | hsa00120 | 46 | 0.27 | 1 | 0.24 | 1 | 1 | 3.7037 | 0.61978876 | | | Cholesterol |
| Aminoacyl-tRNA biosynthesis | hsa00970 | 48 | 0.281 | 1 | 0.249 | 1 | 1 | 3.55872 | 0.60380065 | | | L-Asparagine |
| Purine metabolism | hsa00230 | 65 | 0.381 | 1 | 0.323 | 1 | 1 | 2.62467 | 0.49079748 | | | Guanine |
| Steroid hormone biosynthesis | hsa00140 | 85 | 0.498 | 1 | 0.402 | 1 | 1 | 2.00803 | 0.39577395 | | | Cholesterol |

**Extended Data Table 2. KEGG pathway enrichment analysis**
